# Further characterization of the GlyT-1 inhibitor Org25935: anti-alcohol, neurobehavioral, and gene expression effects

**DOI:** 10.1007/s00702-017-1685-z

**Published:** 2017-02-04

**Authors:** Helga Höifödt Lidö, Susanne Jonsson, Petri Hyytiä, Mia Ericson, Bo Söderpalm

**Affiliations:** 1 Addiction Biology Unit, Department of Psychiatry and Neurochemistry, Institute of Neuroscience and Physiology, Sahlgrenska Academy, University of Gothenburg, Gothenburg, Sweden; 2 Department of Pharmacology, University of Helsinki, Helsinki, Finland; 3 Beroendekliniken, Sahlgrenska University Hospital, Gothenburg, Sweden

**Keywords:** Alcohol use disorder, Glycine, Glycine transporter-1 inhibitors, Ethanol intake, Wistar-rats, AA-rats

## Abstract

The glycine transporter-1 inhibitor Org25935 is a promising candidate in a treatment concept for alcohol use disorder targeting the glycine system. Org25935 inhibits ethanol-induced dopamine elevation in brain reward regions and reduces ethanol intake in Wistar rats. This study aimed to further characterise the compound and used ethanol consumption, behavioral measures, and gene expression as parameters to investigate the effects in Wistar rats and, as pharmacogenetic comparison, Alko-Alcohol (AA) rats. Animals were provided limited access to ethanol in a two-bottle free-choice paradigm with daily drug administration. Acute effects of Org25935 were estimated using locomotor activity and neurobehavioral status. Effects on gene expression in Wistar rats were measured with qPCR. The higher but not the lower dose of Org25935 reduced alcohol intake in Wistar rats. Unexpectedly, Org25935 reduced both ethanol and water intake and induced strong CNS-depressive effects in AA-rats (withdrawn from further studies). Neurobehavioral effects by Org25935 differed between the strains (AA-rats towards sedation). Org25935 did not affect gene expression at the mRNA level in the glycine system of Wistar rats. The data indicate a small therapeutic range for the anti-alcohol properties of Org25935, a finding that may guide further evaluations of the clinical utility of GlyT-1 inhibitors. The results point to the importance of pharmacogenetic considerations when developing drugs for alcohol-related medical concerns. Despite the lack of successful clinical outcomes, to date, the heterogeneity of drug action of Org25935 and similar agents and the unmet medical need justify further studies of glycinergic compounds in alcohol use disorder.

## Introduction

Alcohol use disorder (AUD) is a heterogeneous disorder affecting multiple neurotransmitter systems of which the mesolimbic dopamine system, a part of the reward pathway, still attracts considerable interest. The reward signal induced by alcohol (ethanol), as well as other drugs of abuse, corresponds to a dopamine increase in nucleus accumbens (nAc) (Di Chiara and Imperato 1988; Koob and Bloom 1988; Wise 1996). Over time, chronic ethanol exposure may lead to neuroadaptations in the striatal dopamine systems, which most likely contribute to the development of addiction (Volkow et al. 2007; Koob and Volkow 2010).

The search for improved pharmacotherapy with novel mechanisms of action is an ongoing quest (Heilig and Egli 2006; Lim et al. 2013). One treatment concept under investigation for AUD targets the glycinergic system. Glycine serves dual functions with regards to neurotransmission, as an agonist at inhibitory glycine receptors (GlyRs) and as co-agonist at excitatory *N*-methyl-d-aspartate receptors (NMDARs) (Bowery and Smart 2006; Kirsch 2006). Since both receptor populations are primary targets for alcohol (Yevenes et al. 2008) and can modulate the mesolimbic dopamine system, glycine-based compounds may exert its action by a bimodal mechanism interacting with GlyRs and NMDARs (Molander and Söderpalm 2005a; Spanagel 2011 2009). The biological rationale for glycine signaling as a therapeutic target for AUD originates from animal models demonstrating that GlyRs in the nAc are access points for ethanol to the mesolimbic dopamine system (Soderpalm et al. 2009; Soderpalm and Ericson 2013). Glycine was shown to elevate extracellular dopamine levels in the nAc and decrease ethanol consumption in rats, and conversely, the GlyR antagonist strychnine was found to decrease dopamine in the nAc and increase ethanol consumption (Molander et al. 2005).

**Fig. 1:**
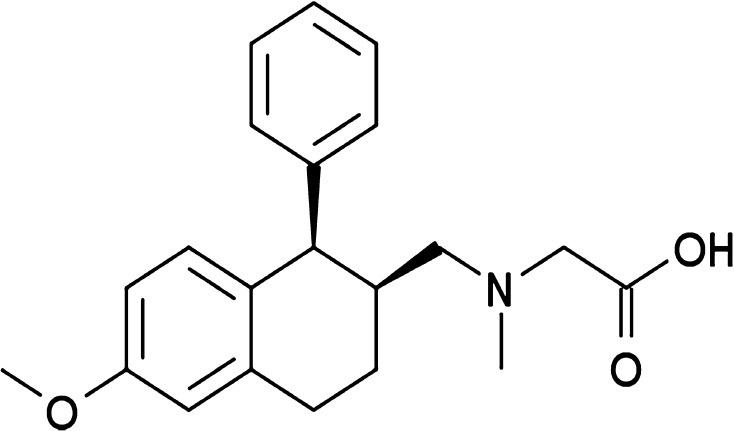
Chemical structure of Org25935. For biochemical properties, see “Introduction” section

The glycine transporter 1 protein (GlyT-1) is the principal regulator of extracellular glycine (Gomeza et al. 2003; Cubelos et al. 2005) and CNS glycine concentrations are elevated by GlyT-1 inhibitors (Betz et al. 2006; Zafra and Gimenez 2008). Several GlyT-1 inhibitors, initially developed for the treatment of schizophrenia, have been investigated with respect to interference with ethanol-induced dopamine elevation as well as consumption. The strongest GlyT-1 inhibitor candidate so far has been Org25935, also known as SCH 900435 (Harvey and Yee 2013). Org25935 (chemical name: cis-*N*-methyl-*N*-(6-methoxy-1-phenyl-1,2,3,4-tetrahydronaphthalen-2-ylmethyl)amino-methylcarboxylic acid; Fig. 1) is a sarcosine-based, non-competitive, and highly selective GlyT-1 inhibitor lacking significant affinity for other transporters and receptors, including those for dopamine, serotonin, noradrenaline, glutamate, and gamma-aminobutyric acid (GABA). Org25935 demonstrates moderate potency (IC50 is 0.162 µmol/L) and high radioligand binding in several brain regions in the rat (Andrews et al. 2007). The compound shows dose-linear pharmacokinetics with dose-dependent selective increase of glycine levels in frontal cortex (Andrews et al. 2007), nAc, and dorsal striatum (Ge et al. 2001). Org25935 was also demonstrated to induce a robust and long-lasting reduction of voluntary ethanol consumption and reverse compulsive relapse-like alcohol-drinking (Molander et al. 2007; Vengeliene et al. 2010). In more detail, administration of Org25936 (6 mg/kg i.p.) evoked an 87% increase of accumbal glycine levels, which, in turn, prevented ethanol from increasing dopamine in the same brain region (Lidö et al. 2009, 2011). These combined effects are believed to underlie Org25935’s therapeutic potential.

The aim of this study was to further characterise Org25935 with respect to neurobehavioral, anti-alcohol, and gene expression effects. Current models of assessing AUD have shown varying predictive validity, and for that reason, it was of interest to add a pharmacogenetic aspect in the evaluation of Org25935 (Egli 2005). Therefore, drug responses were evaluated in Alko-Alcohol (AA) rats, a genetic model for high alcohol preference, in addition to outbred Wistar rats. The AA rat line demonstrates a tenfold higher alcohol consumption compared to its counterpart, the Alko Non-Alcohol (ANA) rat line (Eriksson 1968; Sommer et al. 2006), yet the underlying genetic factors in predilection towards ethanol drinking are not fully understood (Hyytia et al. 1999; Ojanen et al. 2006). First, effects of single doses of Org25935 on locomotor activity and additional 10 neurobehavioral responses were evaluated. Then, effects of repeated dosing of Org25935 were tested in an ethanol consumption paradigm.

Another aim was to determine whether GlyT-1 inhibition would affect regional gene expression related to this treatment. GlyRs are pentameric chloride-conducting channels composed of α1-4 and β subunits, either as 2α3β-heteromers or α-homomers (Grudzinska et al. 2005). Here, expression of genes encoding GlyR subunits α1-3 (Glra1-3) and β (Glrb) and transporters for glycine, i.e., GlyT-1 (Slc6a9) and taurine, TauT, (Slc6a6) were compared between Org25935-treated animals and controls. Effects of Org25935 on glycinergic gene expression have previously been investigated, but only in caudate putamen and in combination with ethanol consumption (Vengeliene et al. 2010). In a wider perspective, effects on amino-acid neurotransmitters by glycine could affect inhibitory signaling in general, making adjustments in the GABAergic system a possible outcome of Org25935-treatment. Therefore, we included genes for GABA transporters (GAT) 1 (Slc6a1) and 3 (Slc6a11) and gephyrin (Gphn), the latter a protein anchoring both GlyRs and GABA_A_Rs to the synapse. Gene expression was monitored in regions involved in reward-related behaviors, i.e., the nAc (also known as ventral striatum), dorsal striatum, prefrontal cortex, and amygdala.

## Materials and methods

### Animals

A total of 80 male Wistar rats were supplied by Taconic (Ejeby, Denmark), whereof 60 for the ethanol consumption study (43 were selected after screening) and 20 for behavioral assessment. Forty AA-rats were kindly provided by Dr Petri Hyytiä (University of Helsinki, Finland), and were used in the ethanol consumption study, whereof 20 were assessed in the behavioral battery. Animals were kept under reversed light–dark conditions (lights off at 7.00 a.m. and on at 7.00 p.m.) at constant room temperature (22 °C) and humidity (65%) with free access to water and standard rat feed (Harlan Teklad Europe, UK). Rats weighed 300–400 g at the beginning of the experiment.

### Drugs

Org25935 (provided by Organon Labs. Ltd., Newhouse, UK, now merged with Merck, Sharp and Dohme, Newhouse, Lanarkshire, UK), was dissolved in 0.9% NaCl and administered i.p. at a volume of 2 ml/kg. The same volume of 0.9% NaCl was used as vehicle. The dose of 6 mg/kg Org25935 was selected based on the previous studies (Ge et al. 2001; Molander et al. 2007; Lidö et al. 2009). The compound was injected 40 min before the test sessions, as extracellular glycine levels are expected to peak 50 min after Org25935 administration (filed data, Organon Labs. Ltd.). In the ethanol consumption study, a 2.5 h fluid consumption period was chosen, since Org25935 increases striatal glycine levels for 2.5 h (Ge et al. 2001). Two 300 mL drinking bottles were available during the drinking session, one with regular tap water and one with ethanol (Kemetyl AB, Haninge, Sweden). The animals were randomly assigned to drug and vehicle groups in all experiments.

### Screening for ethanol preference

During 2 weeks of continuous access to ethanol and water, the ethanol concentration was gradually increased from 2 to 6% for Wistar rats and from 2 to 10% for AA-rats. The 6% ethanol (v/v) solution was used for Wistar rats, since ethanol consumption in this strain is highest at this concentration (Fahlke et al. 1994). A 10% ethanol (v/v) solution was used for AA-rats, since they are selectively bred based on preference for a 10% solution (Eriksson 1968). Animals were individually housed in plastic cages and fluid intake was measured twice a week over a 5-week period. The amount of ethanol solution consumed in percent of total fluid intake was used as ethanol preference index. Rats with >30% were included in the study (71% of Wistar rats reached this criterion, 100% of AA-rats).

### Behavioral activity: limited access drinking experimental procedure

After the initial screening, the animals were placed on a limited access paradigm. During the first week, rats had access to ethanol and water for 2.5 h/day to adapt the animals to the procedure and to establish a baseline limited access drinking prior to the experiment. Rats were subsequently treated with daily Org25935 or NaCl, injected i.p. 40 min before being presented with the choice of ethanol or water (drinking sessions lasted from 10.00 a.m. to 12.30 p.m.). Org25935 at doses of 3 or 6 mg/kg were tested in two distinct sets of Wistar rats. Due to decreased tolerance to the high dose of Org25935 in AA-rats, the dose was lowered to 3 mg/kg 2 days into the experiment.

### Behavioral activity: locomotor activity procedure

The locomotor apparatus consisted of eight sound-attenuated, ventilated, and dimly lit monitoring boxes (700 × 700 × 350 mm). For photocell detection, the boxes were equipped with two levels of five by five rows of photocell beams, placed 10 and 130 mm above the floor level, respectively, and connected to a computer-based system to register the activity of the rats (Kungsbacka mät-och reglerteknik AB, Fjärås, Sweden). Locomotor activity was defined as the accumulated number of new photocell beams interrupted during a 30-min period. Rearing activity was defined as the number of new photocell beams interrupted at the top row of photocells.

### Behavioral activity: the functional observational battery

Neurobehavioral functioning was assessed using selected measures from a previously described functional observational battery (Mattson et al. 1996). The measures were selected based on behavioral observations following administration of Org25935 in the previous experiments. The battery consisted of ten behavioral, neurological, and autonomic measures and rats were tested in the order listed: overall status, respiration, aggressiveness, posture, muscle tone, catalepsy, righting response, rigidity, vocalisation, and piloerection. The criteria for scoring were based on a previous procedure for validation of behavioral activity using categorical scorings (Ingman et al. 2004), and test measures were evaluated by the rank score system (0–2) in Table 1. Animals were assessed six times; 40, 50, 60, 70, 80, and 90 min after receiving Org25935 (6 mg/kg) or NaCl i.p. At each test session, rats were evaluated independently by two operators both blinded to the treatment, and were returned to their home cages between sessions.

**Table 1:** Functional operational battery: assessments and criteria for scoring

Overall status	Rigidity
0 Normal healthy rat	0 Normal
1 Mild deviation from normal status	1 Mild
2 Severe deviation from normal status	2 Severe
Neurologic signs	Behavioral signs
Posture (curved back score)	Aggressiveness
0 Normal	0 None
1 Mild curved back	1 Struggling when handled on a bench
2 Marked curved back	2 Struggling, must be kept in neck grip
Muscle tone	Vocalisation
0 Normal	0 None
1 Somewhat flaccid	1 Some
2 Completely flaccid	2 Marked
Catalepsy	Autonomic signs
0 Forelimb quickly removed from object	Respiration
1 Delayed removal	0 Normal
2 Forelimb remains on object	1 Irregular/dyspnea
Righting response	2 Pronounced dyspnea or wheezing
0 Quick turn (to all paws under body)	Piloerection
1 Slow turn	0 None
2 No turn	1 Mild
	2 Marked

The ten behavioral, neurological, and autonomic measures recorded and the scoring criteria for scores of 0, 1, or 2 are presented. For further description, see “Materials and methods”

### Gene expression: experimental design

Due to the deviant behavior observed following Org25935 challenge, AA-rats did not continue to gene expression analysis, whereas Wistar rats underwent such an analysis, with the aim to explore possible mechanisms underlying the rapid adaptation to the initial CNS-depressive effect (reduced water intake treatment day one and two). Twelve adult male Wistar rats were used for this study. Rats (180–200 g at arrival) were randomly assigned to active treatment (Org25935, *n* = 6) or placebo (NaCl, *n* = 6) and received daily administration (10.00 a.m.) for 19 consecutive days. No signs of intolerability were observed during the treatment. On day 20, animals were decapitated and brains dissected according to Heffner, using a brain matrix and under strictly RNase-free conditions (Heffner et al. 1980). Brains were kept cold during dissection and dissected tissues were collected in RNase-free Eppendorf tubes, frozen on dry ice, and then kept at −80 °C until further processing.

### RNA extraction and cDNA synthesis

RNA extraction and cDNA synthesis were performed as previously described (Jonsson et al. 2009, 2012). Briefly, samples were homogenised in 1-ml QIAzol Lysis Reagent (Qiagen AB, Sollentuna, Sweden) and RNA was extracted using a Qiagen RNeasy kit according to the manufacturer’s protocol (Qiagen). RNA concentrations of all samples were determined with a SmartSpec Plus spectrophotometer (Bio-Rad Laboratories AB, Sweden) and material corresponding to 1-µg RNA was used for cDNA synthesis (QuantiTect Reverse Transcription Kit, Qiagen). Synthesis of cDNA was performed in accordance with the manufacturer’s instructions.

### Real-time quantitative PCR analysis

QuantiTect Primer Assays were used for gene-specific analysis (Qiagen), as previously described (Jonsson et al. 2009). Analyses of expression of reference genes (β-actin, GAPDH, and RPL19) and target genes Glra1, Glra2, Glra3, Glrb, Slc6a9, Slc6a6, Gphn, Slc6a1, and Slc6a11 were carried out in 96-well plates using a LightCycler^®^ 480 Real-Time PCR System (Roche Applied Science). Calibrators and negative controls (in duplicates) were included in every assay. Primer efficiencies were decided by standard curves with reverse-transcribed RNA. A melting curve analysis for each sample was performed to ensure that a single product was obtained. The threshold value, (*C*_t_), for each sample was determined by the LC 480 Software Version 1.5 (Roche Applied Science) and data were exported as text files and imported into Microsoft Excel for further statistical analyses. Normalisation and analyses were performed as previously described (Pfaffl 2001; Jonsson et al. 2009).

### Statistics

Data with continuous variables were statistically analyzed using the analysis of variance (ANOVA) with repeated measures in SPSS (version 17.0 for Windows). Analyses revealing significant main effect or interactions were followed by Fisher’s protected least significant-difference (PLSD) test, paired *t* test within groups, or unpaired *t* tests between groups, as appropriate. The behavioral data with categorized ratings were evaluated by ordinal regression of summary statistics using Proc Logistic (the logistic procedure) in Statistical Analysis System (SAS). Analysis of gene expression was performed using Mann–Whitney tests in SPSS. Grubbs test was used to exclude outliers (GraphPad Software Inc., USA). The ethanol consumption studies using Wistar and AA-rats differed in time and experimental setups and these data sets were accordingly analyzed separately. Results are presented as mean ± standard error of the mean (SEM) and a probability level (*p*) of less than 0.05 was considered significant.

## Results

### Wistar rats—Effects of 6 mg/kg Org25935 on ethanol and water intake

**Fig. 2:**
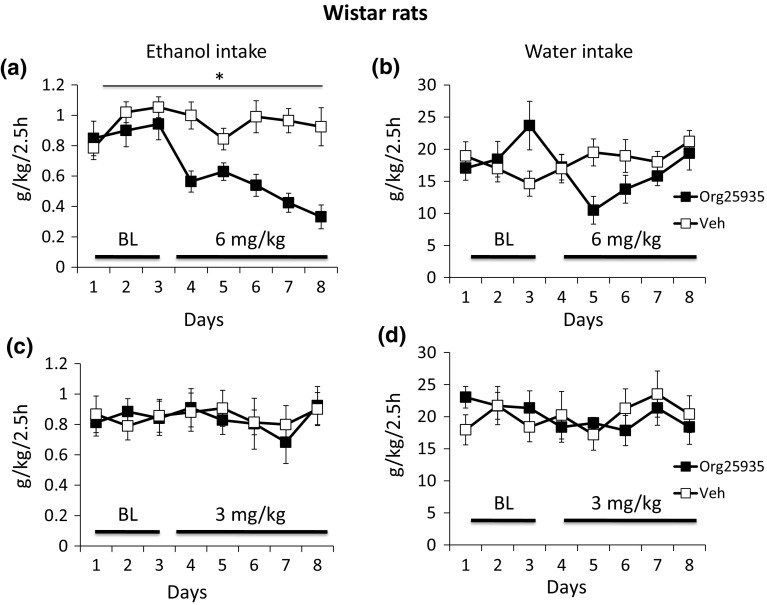
Data from 2.5-h limited access alcohol (6% v/v) drinking in Wistar rats. Displayed are effects on **a** ethanol intake and **b** water intake, *n* = 11–12, after treatment with Org25935 (6 mg/kg i.p.) or vehicle 40 min prior to the drinking session for five consecutive days (d 4–8). The last 3 days of seven baseline days are displayed in the graph (d 1–3) and mean ethanol preference during d 1–3 was 49 and 48% for rats receiving Org25935 and controls, respectively. In the same paradigm, effects of treatment with Org25935 (3 mg/kg i.p.) or vehicle for five consecutive days on **c** ethanol intake and **d** water intake, *n* = 10–11 are displayed. The mean ethanol preference of the last three baseline days (d 1–3) was 47 and 43% for rats receiving Org25935 and controls, respectively. Shown are mean ± SEM. For statistics, see the “Results” section

Treatment with Org25935 for 5 days reduced ethanol intake in comparison with the vehicle-treated group ANOVA over day 1–8 (Fig. 2a) showed a significant effect of treatment [*F*(1,21) = 5.223, *p* = 0.033], time [*F*(7,147) = 6.542, *p* < 0.001] and a treatment × time interaction [*F*(7,147 = 7.409, *p* < 0.001]. The anti-alcohol intake effect was evident from the first day of treatment (*p* = 0.022). Org25935 was also found to have initial effect on the water intake (Fig. 2b) but no statistical effect over the whole treatment period. ANOVA over day 1–8 showed effect of treatment × time [interaction term: *F*(7,147) = 3.092, *p* = 0.005] but no effect of group [*F*(1,21) = 0.510, *p* = 0.483] or time [*F*(7,147) = 1.211, *p* = 0.300].

### Wistar rats: effects of 3 mg/kg Org25935 on ethanol and water intake

Treatment with Org25935 for 5 days did not influence ethanol intake (treatment [*F*(1,20) = 1.000, *p* = 0.329], time [*F*(8,160) = 0.998, *p* = 0.439], treatment × time [*F*(8,160) = 0.997, *p* = 0.441]) or water intake (treatment [*F*(8,17) = 0.106, *p* = 0.748], time [*F*(8,152) = 1.014, *p* = 0.428], and treatment × time [*F*(8,152) = 1.603, *p* = 0.129]) when analyzed by ANOVA over day 1–8 (Fig. 2c, d).

### AA-rats: effects of 6 and 3 mg/kg Org25935 on ethanol and water intake

**Fig. 3:**
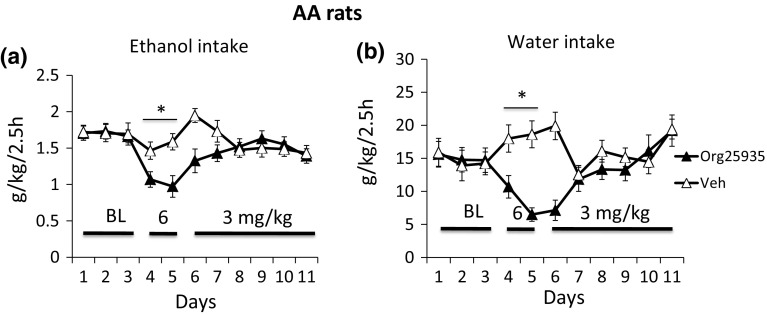
Data from 2.5-h limited access alcohol (10% v/v) drinking in Alko-Alcohol (AA) rats after vehicle or 2 days of treatment with Org25935 6 mg/kg i.p. (d 4–5) and 6 days (d 6–11) with 3 mg/kg i.p. The drug was administered 40 min prior to the drinking session. The last 3 days of the seven baseline days are displayed in the graph (d 1–3) and mean ethanol preference of d 1–3 was 62 and 61% in AA rats receiving Org25935 and controls, respectively. Shown are the mean ± SEM of effects on **a** ethanol intake and **b** water intake in 17–20 animals. For statistics, see the “Results” section

The effect of Org25935 on ethanol intake, dosed at 6 mg/kg for 2 days followed by 3 mg/kg i.p for 6 days, is displayed in Fig. 3a. Due to observed side-effects 2 days into the 6 mg/kg challenge, treatment was discontinued in two rats, while one rat was killed due to intolerable suffering and the dose was lowered in the remaining rats. ANOVA over days 4–5 revealed an effect on group [*F*(1,36) = 8.811, *p* = 0.005] but not time [*F*(1,36) = 0.020, *p* = 0.888] or interaction [*F*(1,36) = 0.922, *p* = 0.343] during the 2 days of 6 mg/kg challenge (Fig. 2a). There was a group effect [*F*(1,36) = 7.071, *p* = 0.012] during the first 2 days of 3 mg/kg (day 6 and 7 in Fig. 2a) but no time effect [*F*(1,35) = 0.186, *p* = 0.669] or interaction term effect [*F*(1,35) = 2.302, *p* = 0.138]. Ethanol intake was significantly reduced compared to mean baseline level on day 4 (*p* = 0.002) and day 5 (*p* = 0.002) but returned to baseline levels on day 6. Analysis of water intake revealed a significant effect of time [*F*(10,290) = 2.306, *p* = 0.013] and interaction [*F*(10,290) = 5.130, *p* < 0.001] but not group [*F*(1,29) = 3.564, *p* = 0.069] over the whole time period. Over day 4 and 5, (see Fig. 3b) ANOVA revealed an effect of 6 mg/kg on water intake [*F*(1,34) = 24.753, *p* < 0.001] but no effects of time [*F*(1,34) = 1.301, *p* = 0.262] or interaction [*F*(1,34) = 2.281, *p* = 0.140]. A difference between treated animals and controls was also observed on day 6 and 7, the first 2 days of the 3 mg/kg dose regimen [*F*(1,32) = 13.439, *p* = 0.001], time [*F*(1,32) = 4.612, *p* = 0.039], and interaction term [*F*(1,32) = 27.049, *p* < 0.001].

### Motor activity

**Fig. 4:**
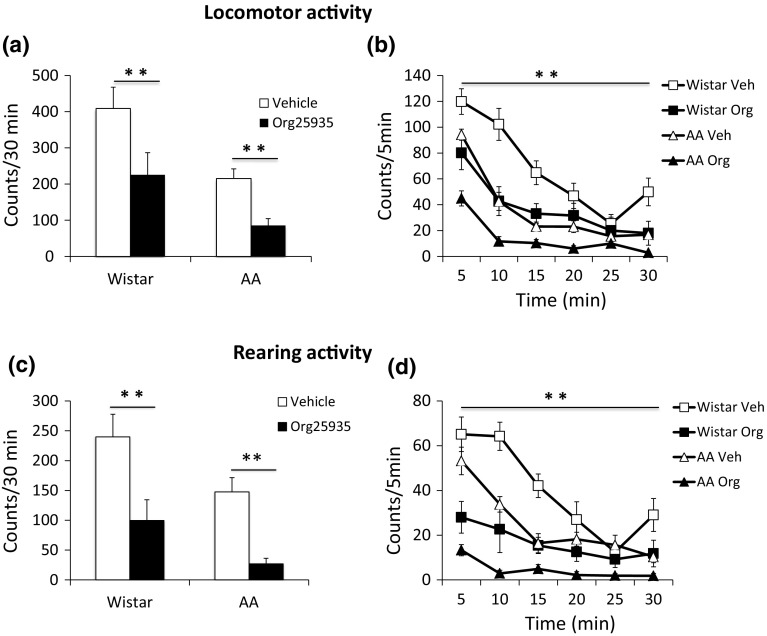
Effect of Org25935 (6 mg/kg i.p.) or vehicle on locomotor activity. Results are displayed as accumulated counts during the entire 30-min period (**a**) and as effect over time, where counts every 5 min were recorded for 30 min (**b**). Shown are the mean ± SEM, *n* = 10. For statistics, see the “Result”. Effect of Org25935 (6 mg/kg i.p.) or vehicle on rearing activity. Results are displayed as accumulated counts during the entire 30-min period (**c**) and as effect over time where counts every 5 min were recorded for 30 min (**d**). Shown are the mean ± SEM, *n* = 10. For statistics, see the “Results”

Wistar rats displayed a higher baseline motor activity as compared to AA-rats and Org25935 significantly reduced motor activity in both strains, yet there were no treatment-related differences in motor activity between the strains. Acute Org25935 suppressed locomotor (Fig. 4a, b) and rearing activity (Fig. 4b, c) in both strains. A pattern of habituation with an initially high motor activity, which declined under the test session, was evident in both strains. ANOVA revealed an overall effect of treatment [F(1,36) = 25.252, *p* < 0.001] and strain [F(1,36) = 28.653, *p* < 0.001] on locomotor activity, but treatment effects did not differ between strains [F(1,36) = 0.736, *p* = 0.397]. Similarly, for rearing activity, an overall effect of treatment [F(1,36) = 46.603, *p* < 0.001] and strain [F(1,36) = 18.609, *p* < 0.001] was observed, but no difference of treatment between strains [F(1,36) = 0.269, *p* = 0.607].

### Behavioral and neurological measures

The scoring criteria for scores of 0, 1, or 2 are presented in Table 1. The maximum group score possible per session was 20 (*n* = 10/group). Each rat was evaluated at six time-points, at 40, 50, 60, 70, 80, and 90 min after drug administration (time wise scores are not shown). The total group scores and number of scoring rats per group are presented in Table 2, where asterisk indicates significant *p* values. Overall status: A score was given if behavior or status differed from healthy naïve rats. Ordinal regression revealed a significant effect of treatment (*p* < 0.001), but not of ratline or interaction treatment × ratline. Respiration: No significant differences in respiration were found. Two AA-rats receiving Org25935 demonstrated dyspnoea and wheezing symptoms after 60 min. Aggressiveness: A trend towards effect of ratline (*p* = 0.080) but not for treatment or interaction treatment × ratline (*p* = 0.452) was found. Four Org25935-treated Wistars, as compared to one rat in other treatment groups, displayed aggressive behavior. Body posture: The body posture was measured based on the curvature of the back and effect of treatment was manifested in both strains (*p* < 0.001) but not of ratline or interaction treatment × ratline. None of the measures muscle tone, catalepsy, righting response, muscle tone, stiffness, vocalisation, or piloerection displayed any differences.

**Table 2:** Functional operational battery: Score results

Treatment group	Total group score	Number of scoring rats	Total group score	Number of scoring rats
	Overall status		Respiration	
AA-Vehicle	14	3	0	0
AA-Org25935	66*	7	11	2
Wis-Vehicle	6	3	0	0
Wis-Org25935	59*	9	0	0
	Aggressiveness		Body posture	
AA-Vehicle	1	1	12	2
AA-Org25935	2	1	67*	7
Wis-Vehicle	5	1	5	5
Wis-Org25935	13	4	55*	9
	Muscle tone		Catalepsy	
AA-Vehicle	0	0	0	0
AA-Org25935	41	4	28	5
Wis-Vehicle	2	1	0	0
Wis-Org25935	9	2	6	2
	Righting response		Rigidity	
AA-Vehicle	0	0	5	2
AA-Org25935	39	4	19	5
Wis-Vehicle	0	0	0	0
Wis-Org25935	3	1	9	3
	Vocalisation		Piloerection	
AA-Vehicle	11	2	0	0
AA-Org25935	0	0	0	0
Wis-Vehicle	14	4	0	0
Wis-Org25935	31*	8	0	0

Effects of acute Org25935 (6 mg/kg i.p.) and vehicle on the neurobehavioral measures in Wistar and AA rats (*n* = 10). The total group scores and number of scoring rats per group are presented in Table 2, where * indicates significant *p* values. * *p* < 0.05 For further statistics, see the “Results” section

To further analyze differences in treatment response between the strains, Wistar rats and AA-rats were analyzed separately. For overall status and body posture, a significant treatment effect was observed in both AA-rats (*p* < 0.001) and Wistars (*p* < 0.001). There was a trend towards treatment effect on righting response in AA-rats (*p* = 0.0865) but not in Wistars (*p* = 0.9374). Org25935 increased vocalisation in Wistar (*p* = 0.032) but not in AA-rats. Trends towards an effect of Org25935 treatment in AA-rats on muscle tone and righting response were observed. The total score for muscle tone was 41 in AA versus 10 in Wistar rats, whereas total score for righting response was 39 in AA versus 3 in Wistar rats receiving Org25935.

### Gene expression

**Fig. 5:**
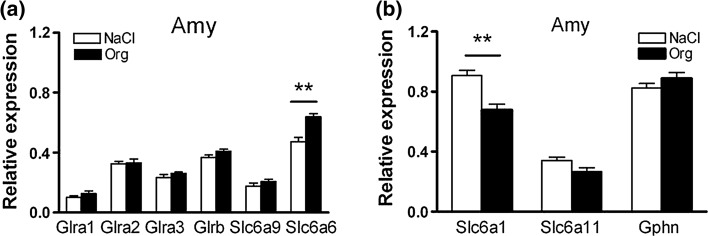
Gene expression in Amygdala of genes involved in glycinergic transmission. Fig a displays Glra1 (GlyR subunit α1), Glra2 (GlyR subunit α2), Glra3 (GlyR subunit α3), Glrb (GlyR subunit β), Slc6a9 (Glycine transporter 1), and Slc6a6 (Taurine transporter). Fig b displays Slc6a1 (GABA transporter 1), Slc6a11 (GABA transporter 3), and Gphn (gephyrin). Treatment with Org25935 for 19 consecutive days did generally not affect expression of glycinergic genes. Exceptions were an up-regulation of Slc6a6 (TauT) (**a**) and a down-regulation in Slc6a1 (GAT-1) gene expression (**b**) in Org25935-treated rats. Shown are the mean ± SEM, *n* = 6. **p < 0.01. Statistics, see “Results”. Negative results from the other brain regions are not presented

Mann–Whitney tests used to compare the relative gene expression between Org25935-treated Wistar rats and controls revealed only two significant differences in gene expression, both in the amygdala (Fig. 5). In this region, Org25935-treatment resulted in up-regulation of Slc6a6 (TauT, *p* = 0.004) and down-regulation of Slc6a1 (GAT-1, *p* = 0.009). A trend towards down-regulation was also observed in Slc6a11 expression (GAT-3, *p* = 0.065). Another trend, also towards down-regulation in Org25935-treated animals, was observed for Glra2 in nAc *p* = 0.065, data not shown). No differences in gene expression between active group and control group were observed in any of the brain regions nAc, prefrontal cortex, or dorsal striatum.

## Discussion

This study demonstrates an anti-alcohol-drinking effect of Org25935 (6 mg/kg) in Wistar rats, even though the drug initially tended to decrease also water intake, which was then normalized. This confirms the previously reported alcohol-sparing effect of Org25935 observed in high (≥60% preference for ethanol) and medium (30–60% preference for ethanol) alcohol-preferring rats (Molander et al. 2007) and in rats with relapse-like drinking behavior (Vengeliene et al. 2010). That another sarcosine-based non-competitive GlyT-1 inhibitor possesses similar properties supports a class—rather than a substance-bound effect (Lifö et al. 2011). Org25935 has demonstrated long-lasting alcohol intake reducing properties in rodent models with effects superior to most drug candidates for AUD (Spanagel and Kiefer 2008). The compound has a good safety profile and neither animal investigations nor human studies indicate a positive hedonic profile (Liem-Moolenaar et al. 2013). However, the attempt to translate these promising results to AUD patients failed; the proof of concept trial was aborted before completion due to futility in the interim analysis (see last section).

To our knowledge, this is the first report of a GlyT-1 inhibitor in AA-rats. The obtained results were unexpected; Org25935 induced an abrupt CNS depression in AA-rats, reduced both water and ethanol intake, and caused two mortalities by respiratory depression on day 2 of treatment. The symptoms disappeared with dose reduction, but no specific effect on ethanol consumption was revealed. This prevented a sound evaluation of the drug’s anti-alcohol-drinking potential in this strain (see “Discussion”, section five). Distinctions in outcomes across rat strains are a known phenomenon (Egli 2005). Motor and respiratory depression for GlyT-1 inhibitors in rodents has been reported previously, and these effects were attenuated by the GlyR antagonist strychnine, pointing to involvement of the GlyR (Perry et al. 2008). That the CNS symptoms were more pronounced in inbred AA-rats may suggest that the low dose tolerability has a background in glycinergic genes. Other possibilities for the differential sensitivity between strains could be different Org25935 pharmacokinetics, or that the larger alcohol intake made AA-rats more sensitive due to synergistic CNS-depressive effects between Org25935 and ethanol. Whatever the reason is, the motor and respiratory depression hampered a meaningful preclinical evaluation in the AA-rats. Caution must be taken when utilizing genetic models for generating a certain phenotype and pharmacogenetics should be considered in the development and of new drug candidates for AUD.

In this study, Org25935 in the dose 3 mg/kg (raising extracellular glycine levels by 25%) did not influence ethanol consumption. Org25935 passes the blood–brain barrier and systemic administration of 3, 6, or 10 mg/kg i.p. elevates dialysate glycine levels by 25, 80, and 130%, respectively (Ge et al. 2001). GlyT-1 blockade at 6 mg/kg elevated accumbal glycine output by 85% and counteracted ethanol-induced dopamine release in rat nAc (Lidö et al. 2009, 2011). Assuming that the interference with ethanol-induced dopamine elevation was, at least partly, related to the alcohol-sparing effect, the dose of 6 mg/kg was selected for further behavioral assessments. The lack of effect on alcohol intake with the lower dose is not in agreement with results from rats with compulsive drinking following long-term ethanol exposure. In these rats, 3 mg/kg demonstrated anti-alcohol-drinking effect (Vengeliene et al. 2010). This could reflect a neurobiological adaptation in glycinergic mechanisms following chronic ethanol.

The lack of effect of the lower dose of 3 mg/kg (lower GlyT-1 occupancy) indicates a narrow dose range for Org25935. Data from non human primates suggest an inverted-U dose-response curve for cognitive performance (traditionally thought to be mediated via NMDARs rather than GlyRs), indicating a complexity in the CSF glycine level-efficay relationship (Castner et al. 2014). An apparent bell-shaped dose–efficacy relationship is seen also for other GlyT-1 compounds and suggests that the optimal pharmacokinetic level is in a narrow range (Eddins et al. 2014). GlyT-1 inhibitors have been shown to lose their pro-cognitive effects following GlyT-1 occupancy above 50% due to NMDAR endocytosis (Harsing et al. 2003, 2006; Umbricht et al. 2014). Due to the dual receptor binding of glycine and the lower affinity to the GlyR compared to the NMDAR, effect responses on alcohol intake may, perhaps, occur at a distinct dose range as compared to target responses in schizophrenia. As a comparison, effective treatment using GlyT-1 inhibition with respect to cognitive facilitation translates to an approximate 200–250% increase in striatal glycine (Alberati et al. 2012). GlyT-1 pharmacotherapy may require improved pharmacokinetics and/or individualized dosing to yield an inhibitory/excitatory balance needed for anti-alcohol efficacy.

In the locomotor assessments, Wistar rats demonstrated higher baseline locomotion compared to AA-rats. Low baseline motor activity in AA-rats could imply decreased dopamine function, a phenomenon previously associated with addiction and which could drive alcohol intake (Volkow et al. 2007). Yet, neurobiological alterations underlying alcohol preference in AA-rats are mostly found in opioid and endocannabinoid mechanisms and not in the dopamine system (Hyytia 1993; Hyytia et al. 1999; Sommer et al. 2006). Acute Org25935 reduced locomotor activity to the same extent in both strains. As discussed above, rodents are known to respond to GlyT-1 blockade with motor and respiratory side-effects which have been attributed to sustained glycine elevation requiring repeated dosing (Hashimoto 2011). This may explain why AA tolerated the single dose (neurobehavioral study) but not repeated dosing (consumption study). Kopec et al. reported hyper-locomotion and compulsive walking after GlyT-1 compounds with high residence time in mice (Kopec et al. 2010). A speculation explaining the hypo- vs hyper-locomotion is that the previously described ataxia (Perry et al. 2008) is expressed to a greater extent in rats, whereas compulsive walking is expressed more in mice. A stiff and curved back was observed in rats receiving the GlyT-1 inhibitor, a phenomenon in concordance with the previous studies and most likely related to the high density of GlyRs in the rat brain stem (Perry et al. 2008). More AA-rats responded with rigidity, respiratory depression, reduced muscle tone, and weakened righting response. Again, this pattern of CNS depression may indicate pre-existing abnormalities in glycinergic neurotransmission in AA-rats.

Yet, there is a report of minimal quantitative differences between mRNA expression of the GlyR subunits in AA and ANA rats (Jonsson et al. 2009). Accordingly, differences between AA and ANA rats may lie elsewhere in the neuronal circuitry, in GlyT mechanisms, or in mechanisms downstream to GlyR activation. That glycine directly activates the GABA_A_ receptor at high doses could also account for the CNS depression. Indeed, more pronounced sedation and muscle relaxation are observed after benzodiazepines in AA-rats, compared to ANA rats (Ingman et al. 2004), i.e., favouring a GABA signaling alteration in AA-rats. Benzodiazepines are connected to alcohol intake, where low and high doses may increase and decrease alcohol consumption, respectively (Soderpalm and Hansen 1998).

Due to the intolerability AA-rats were withdrawn from further experiments and were not part of the experimental program when the chronic effects of Org25935 (daily for 19 days) on GABA, GlyT-1, TauT, and GlyR subunit mRNA expression were examined. We hypothesized that GlyT-1 blockade would affect gene expression directly related to inhibitory transmission and result in changes in GlyR and glycine-related mRNA expression, but the gene expression study carried out in Wistar rats found that the rapid tolerance development to the initial CNS depression in Wistar rats could not be explained by adaptations in the glycine system. That Org25935 did not evoke changes in either GlyR subunits or GlyT-1 mRNA expression implies that the anti-alcohol effect of Org25935 does not involve general changes in expression of these glycine-related genes. The robustness of glycinergic gene expression is in line with results from the previous studies investigating effects of both selective breeding and alcohol exposure on the glycinergic system (Jonsson et al. 2009, 2012). In a compulsive alcohol-drinking model following 1 year of alcohol exposure, the anti-alcohol properties of Org25935 were manifested for at least 6 weeks during a treatment-free period (Vengeliene et al. 2010). Chronic alcohol produced alterations in expression of glycine and glutamate signaling-related genes, which Org25935 reversed to levels found in ethanol naïve controls, but no GlyT-genes were affected by alcohol. Subsequently, Org25935 treatment reversed many genes affected by ethanol, but not the GlyR subunit genes further emphasizing the robustness of the system. It is, however, possible that chronic Org25935 affects the glycinergic and/or the GABAergic system in other brain regions or via other processes, e.g., protein synthesis, degradation, or other factors downstream of, or parallel to, direct GlyR actions. It is noteworthy that the pattern is distinct from the rapid adaptations known to occur in the GABAergic system after alcohol exposure, such as for, e.g., ‘the one glass of wine’ receptor (Olsen et al. 2007). For review of GABA_A_R and alcohol effects, the reader is referred to Krystal et al. (2006).

Other transporters relevant for inhibitory signaling (presumably affected by Org25935) were also included in the study, but alterations in transporter gene expression were only observed in amygdala for both TauT (Slc6a6) and GAT-1 (Slc6a1). Taurine is structurally similar to GABA and has been shown to exert some of its effects via GABA_A_Rs, as well as mimicking GABA effects (Olive 2002), i.e., Org25935 could, via changes in taurine levels, contribute to these alterations. However, taurine levels appear not to be affected by Org25935 (Ge et al. 2001; Lidö et al. 2009), and expression of Slc6a6 and Slc6a1 was altered in opposite directions. A limitation in the design is that amygdala was not divided and analyzed in substructures, which could have helped explaining the opposing alternations. That the effects on transporters were observed exclusively in the amygdala is interesting, since this region is implicated in different aspects of alcohol-related behavior and dependence (Roberto et al. 2010, 2012). Further studies are needed to investigate the potential involvement of TauT, GAT-1, as well as the amygdala as a target region in the effects of Org25935.

In sum, the study repeats the robust alcohol reducing effect of Org25935 in Wistar rats, yet only with the high dose regimen, pointing to a narrow therapeutic range. The previous preclinical studies justified a Proof of concept study and Org25935 was rapidly tested in AUD patients, allowing minimal time to address translational issues (De Bejczy et al. 2014). Org25935 demonstrated no benefit over placebo in the interim analyses and the trial was stopped due to futility. However, a number of limitations of the study may have influenced the outcome, e.g., the lack of an objective marker for alcohol intake (Walther et al. 2015). Furthermore, the single-dose design may have hindered the subjects to achieve a therapeutic drug level. Observations from our own study site indicated that patients initially responding with characteristic glycine-related side-effects, like visual disturbances, reduced their alcohol intake. This may indicate requirement of a substantial glycine elevation to yield anti-alcohol efficacy. Alternatively, responding individuals may be those with low baseline glycine levels, in whom there is more scope for elevating receptor occupancy (Heresco-Levy et al. 1999). This could then be a parallel to preclinical findings, indicating that only a subgroup of rats responds with a dopamine elevation after glycine elevation with Org25935 (Molander and Söderpalm 2005b; Lidö et al. 2009). That GlyT-1 inhibitors modulate separate neurotransmitter systems (glycine, dopamine, and glutamate) further complicates the picture. In sum, both the present and previous data point to a heterogeneity of drug action of Org25935 (and similar compounds). It is possible that therapeutic effects may be delivered if a responding phenotype can be identified and/or if acceptable pharmacokinetics can be obtained. In conclusion, despite the lack of a successful clinical outcome, to date, the unmet medical need in AUD justifies further studies of glycinergic compounds and hopefully development of a new generation of GlyT-1 uptake inhibitors.

## Abbreviations

AA: Alko Alkohol rats
ANA: Alko non-alcohol rats
AUD: Alcohol use disorder
nAc: Nucleus accumbens
GABA: Gamma aminobutyric acid
NMDAR: *N*-methyl-d-aspartate receptors
GlyR: Glycine receptor
GlyT-1: Glycine transporter protein 1
